# Insight into the cellular fate and toxicity of aluminium adjuvants used in clinically approved human vaccinations

**DOI:** 10.1038/srep31578

**Published:** 2016-08-12

**Authors:** Matthew Mold, Emma Shardlow, Christopher Exley

**Affiliations:** 1 The Birchall Centre, Lennard-Jones Laboratories, Keele University, Keele, Staffordshire, ST5 5BG, UK

## Abstract

Aluminium adjuvants remain the most widely used and effective adjuvants in vaccination and immunotherapy. Herein, the particle size distribution (PSD) of aluminium oxyhydroxide and aluminium hydroxyphosphate adjuvants was elucidated in attempt to correlate these properties with the biological responses observed post vaccination. Heightened solubility and potentially the generation of Al^3+^ in the lysosomal environment were positively correlated with an increase in cell mortality *in vitro*, potentially generating a greater inflammatory response at the site of simulated injection. The cellular uptake of aluminium based adjuvants (ABAs) used in clinically approved vaccinations are compared to a commonly used experimental ABA, in an *in vitro* THP-1 cell model. Using lumogallion as a direct-fluorescent molecular probe for aluminium, complemented with transmission electron microscopy provides further insight into the morphology of internalised particulates, driven by the physicochemical variations of the ABAs investigated. We demonstrate that not all aluminium adjuvants are equal neither in terms of their physical properties nor their biological reactivity and potential toxicities both at the injection site and beyond. High loading of aluminium oxyhydroxide in the cytoplasm of THP-1 cells without immediate cytotoxicity might predispose this form of aluminium adjuvant to its subsequent transport throughout the body including access to the brain.

Aluminium based adjuvants (ABA) are included in human vaccinations to boost or potentiate the immune response, to the injected antigen^1^. Whilst a consensus upon the immunomodulatory mechanism of action of ABA has yet to be reached, it has become increasingly recognised that activation of the innate immune response is crucial for increased antibody titres^1^,^2^. The continued and widespread use of ABA has followed the emergence of recombinantly expressed protein antigens of high purity as a safer alternative to inactivated or attenuated pathogens^2^,^3^. Owing to the homogeneity and generally weak immunogenicity of recombinant antigens, the inclusion of adjuvants is often necessary for the induction of robust immune responses and effective immunisation^2^,^4^. Furthermore, the use of adjuvants in human vaccinations has been linked to adverse effects^5^,^6^ often classified under Autoimmune (or autoinflammatory) syndrome induced by adjuvants (ASIA)^7^. Combined with the relatively low cost of hydrated colloidal aluminium salts and their ease of inclusion as effective adjuvants within clinically approved vaccine formulations, the continued use of ABA in human vaccinations is likely to continue^1^,^2^,^4^.

Of the most commonly used ABA in clinically approved vaccine formulations are the commercially available aluminium oxyhydroxide based, Alhydrogel^®^ and aluminium hydroxyphosphate based, Adju-Phos^®^, adjuvants^8^. More recently, a sulphated derivative of the latter in the form of aluminium hydroxyphosphate sulphate has been used as a single component of an adjuvant system against human papilloma virus (HPV)^9^. Typically, the adsorptive capacity of an ABA to its antigen, dictates its choice in studies of adjuvanticity. In this respect, the choice of adjuvant is selected according to its zeta potential or surface charge^1^ of which Alhydrogel^®^ is positively charged at neutral pH and suitable for adsorption to negatively charged antigens, conversely to negatively charged particulates of Adju-Phos^®^ 1,10. Ovalbumin is frequently used as a model protein antigen in experimental vaccine formulations and owing to its number of side-chain carboxyl groups, possesses a net negative charge^1^,^3^,^10^,^11^. As such, Alhydrogel^®^ continues to predominate as the clinically relevant adjuvant of choice in these studies.

Few studies have employed direct comparative assessments of the physicochemical properties of clinically used ABA formulations with their resultant cellular uptake. Of those studies performed, Flarend and co-workers (1997) revealed the same biodistribution of ^26^Al labelled Alhydrogel^®^ and Adju-Phos^®^ adjuvants, when injected intramuscularly (i.m.) in New Zealand White rabbits^12^. Mass spectrometry analyses of digested tissues identified higher aluminium concentrations in the kidneys with the lowest concentration found in the brain. The dissolution of Adju-Phos^®^ however, was found to be more rapid than that for Alhydrogel^®^ with higher ^26^Al concentrations noted in the surrounding tissues, probably owing to the amorphous nature of the former^12^. Whilst elevated ^26^Al concentrations were found in both urine and tissue samples of those rabbits injected i.m. with Adju-Phos^®^, qualitative data pertaining to their retention and *in vivo* localisation through for example, histological staining methods, were not fully addressed in this study^12^.

More recently, the factors affecting the adsorption of Alhydrogel^®^ and Adju-Phos^®^ to their protein antigen have been considered in simulated vaccine formulations^13^. Interestingly, Alhydrogel^®^ was found to possess a greater adsorptive capacity to its conversely negatively charged protein antigen, in comparison to Adju-Phos^®^. The authors noted that the capacity of Alhydrogel^®^ to adsorb to protein antigens via ligand exchange in addition to electrostatic forces of attraction, explained the greater adsorptive capacity of the adjuvant^13^. Upon the administration of either adjuvant into the injection site however, the physiological milieu encountered is of far greater complexity than that of a vaccine formulation^4^. Weak electrostatic forces of attraction between ABAs and antigens result in the rapid dissolution of the antigen in muscle interstitial fluid (MIF)^4^. The resultant species formed including the free antigen, free particulates of the ABA, particulate ABAs co-adsorbed to the antigen and the soluble species of aluminium, Al^3+^_(aq)_^4^ are crucial determinants of the resultant immune response^4^,^13^,^14^,^15^. As such, establishing the potential routes of cellular uptake and subsequent trafficking of these immune agonists through draining lymph nodes^2^, will shed light on their ability to potentiate the immune response, at sites distant to the injection site^4^,^10^.

Until recently, the unequivocal cellular uptake of ABA had remained elusive, whereby lumogallion [4-chloro-3-(2,4-dihydroxyphenylazo)-2-hydroxybenzene-1-sulphonic acid] was found to act as a selective molecular probe for aluminium^16^. The use of lumogallion has demonstrated the unequivocal identification of aluminium oxyhydroxide-based adjuvant formulations, including Alhydrogel^®^ within a monocytic T helper 1 (THP-1) cell line^16^ and was hence used herein for the unequivocal identification of aluminium. All pathways of endocytosis result in the cellular internalisation of ABA particles via the various routes through cell membranes, ultimately resulting in their presence within lysosomal compartments in cell cytosol^17^.

Phagocytosis is one of the most recognised pathways that governs the cellular internalisation and subsequent degradation of micron-sized particulates of ABAs, through the molecular process of autophagy^17^,^18^. In the latter stages of autophagy and most notably for macroautophagy^19^, the maturation of autophagosomes into autolysosomes acidifies the resultant vesicular compartments formed to approximately pH 4.0 to 4.5^18^. This results in the degradation of internalised particulates of the ABA, thereby releasing Al^3+^_(aq)_ into cell cytosol^4^,^17^,^18^. The release of the enzyme cathepsin B as an endogenous danger signal, in combination with the degradative products of ABAs as damage associated molecular patterns (DAMPs) are thereby suggested to trigger localised inflammation at the injection site^20^. Current understanding of the mechanisms of action of ABA have focused upon the adsorptive capacity of adjuvants in enhancing delivery of the antigen to antigen presenting cells^10^,^21^, activation of inflammasome multi-protein complexes^3^,^22^ and the release of pro-inflammatory cytokines^1^,^10^,^23^ polarising T_H_2 immune responses^1^,^2^,^10^,^22^. In the absence of data following the cellular trafficking and fate of the aluminium adjuvant component of a vaccine, their resultant effects underlying their immunostimulatory properties, remain to be elucidated. Furthermore, the continued use of equivocal methods for the identification of aluminium including the use of the fluorophore morin in the absence of appropriate controls^24^, is likely to give misleading information on the cellular fate of label-free aluminium adjuvants in human vaccinations.

Herein, we draw the direct comparative assessment of the physicochemical factors affecting the unequivocal cellular uptake of the clinically relevant Alhydrogel^®^ and Adju-Phos^®^ adjuvants. Furthermore, the cellular fate of the chemically different adjuvants were established in a relevant *in vitro* T helper 1 (THP-1) cell model of vaccination. Whilst the term ‘alum’ refers to potassium aluminium sulphate (KAl(SO_4_)_2_·12H_2_O), its use is widespread within the literature. As such, ‘alum’ is often used incorrectly to describe all variations of ABA^8^. ABA are neither chemically, physically nor biologically equivalent and differ significantly from non-aluminium adjuvants such as silica or uric acid. In this respect, we additionally investigated the physicochemical properties including particle size and charge of an experimental crystalline magnesium hydroxide and amorphous aluminium hydroxycarbonate adjuvant, Imject™ Alum. Taken collectively, our results support the migratory capabilities of ABA as potential drivers of immunopotentiation, *in vivo*.

## Results

### Zeta potential characterisation of aluminium adjuvants within R10 culture medium

To investigate any potential interactions occurring at the surface interface of Alhydrogel^®^ and Adju-Phos^®^ adjuvants, each was introduced into R10 culture medium and their zeta potential measured. The surface potential of both adjuvant formulations were found equivalent following initial administration into culture medium (−12.88 ± 1.11 and −13.56 ± 1.14 mV, respectively, P > 0.1) and remained stable over 24 h (see Supplementary Fig. 1). The zeta potential values were found significantly dissimilar to those obtained for the vaccine preparations (see Supplementary Table 1), although the difference was more pronounced for positively charged Alhydrogel^®^ (P < 0.01).

### Particle size characterisation of aluminium adjuvants within R10 culture medium via dynamic light scattering, selective filtration and graphite furnace atomic absorption spectroscopy

**Figure 1:**
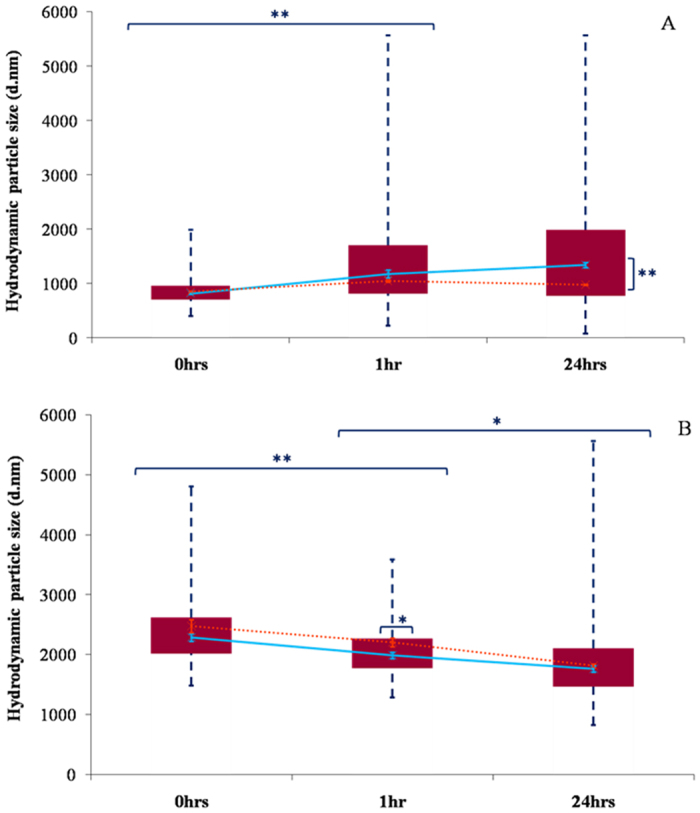
Size distributions of Alhydrogel^®^ (**A**) and Adju-Phos^®^ (**B**) in R10 medium following 0, 1 and 24 h incubation (37 °C). Box plots are representative of the interquartile range of the data while blue dashed lines indicate the maxima and minima. Orange crosses indicate Z-average cumulant size values (nm) while light blue crosses represent the median peak size value (nm). Error bars represent the ±SE of the measurement where *n* = 5.

**Figure 2:**
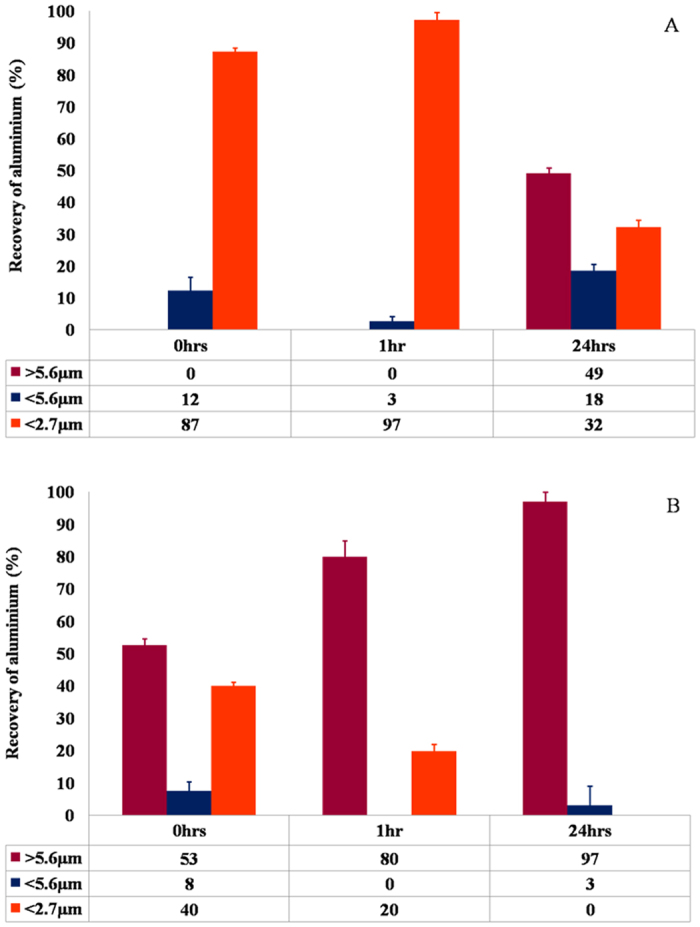
Recovery of Al (%) following selective filtration of Alhydrogel^®^ (**A**) and Adju-Phos^®^ (**B**) after 0, 1 and 24 h incubation in R10 medium (37 °C). Error bars represent the %RSD of the measurement where *n* = 5. Statistical significance not shown.

To assess the impact of protein adsorption upon the size of adjuvant particles within biological media, dynamic light scattering (DLS) was used in conjunction with selective filtration and transversely heated graphite furnace atomic absorption spectroscopy (TH-GFAAS). The latter method provided data regarding the abundance of discrete populations within such polydisperse solutions. Samples of R10 medium inoculated with adjuvanted vaccine preparations produced exclusively monomodal intensity distributions in which the majority of particles formed micron-sized aggregates. The median size of Alhydrogel^®^ aggregates continued to increase over a 24 h incubation period (817.0 ± 19.0 to 1343.0 ± 46.0 nm over 24 h), although this was only significant over the first hour of exposure to culture medium indicating that the particles had reached their maximum size shortly after introduction (Fig. 1a) (P < 0.01). Indeed, the aluminium recovery between the 0.25 and 2.7 μm size exclusion boundaries represented that with the highest magnitude and exhibited a marginal increase over the first hour of incubation (Fig. 1c) (87.1 ± 1.3 and 97.1 ± 2.4% respectively). DLS, however, failed to accurately detect the aggregation occurring following 24 h exposure to R10 medium, as demonstrated by the marked increase in aluminium recovery observed >5.6 μm (Fig. 2a).

Adju-Phos^®^ particulates while larger demonstrated a gradual reduction in median size over 24 h (2283.0 ± 62.0 to 1760.0 ± 52.0 nm), which suggested that disaggregation was occurring within the samples over the course of the experiment (Fig. 1b). However, the highest recovery of aluminium was detected in the filtrates representing particles >5.6 μm and this abundance only increased over the duration of the experiment (Fig. 2b) (52.5 ± 2.0 to 79.8 ± 5.0%). The greater sedimentation rate of larger particles can result in their non-detection via DLS and hence may explain the discrepancies between the results generated by these two different methodologies.

### Transmission electron microscopy of aluminium adjuvants within R10 culture medium

**Figure 3:**
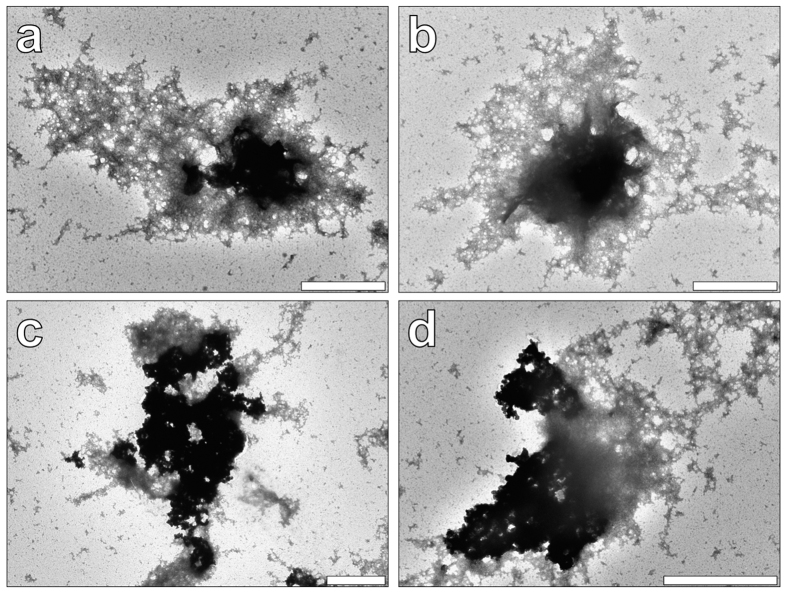
Electron micrographs depicting the morphology of ABA particulates in the presence of R10 culture medium. (**a**,**b**) Alhydrogel^®^ (0.25 mg/mL Al) following 0 h incubation in R10 medium. Magnification X 30 K, scale bars 1 μm. (**c**–**d**) Adju-Phos^®^ (0.25 mg/mL Al) following 0 h incubation in R10 medium. Magnification X 10 K and X 25 K, scale bars 2 and 1 μm, respectively.

In order to confirm the presence of larger adjuvant aggregates unable to be accurately measured by DLS, transmission electron microscopy (TEM) was performed upon vaccine preparations exposed to R10 medium at 0 h. Alhydrogel^®^ particulates ranged from *ca* 1.2–2.0 μm and were therefore found to be in close agreement with those obtained via DLS and selective filtration. The majority of particles identified were found surrounded by an extensive protein corona, which approximately tripled the size of those entities observed (Fig. 3a,b). Conversely, Adju-Phos^®^ particulates were typically much larger in size than those detected via DLS although coronal formation was less extensive, and hence was found to lesser impact upon protein adsorption upon the size of those resultant complexes formed (Fig. 3c,d).

### The cytotoxicity of aluminium adjuvants in a monocytic T helper 1 cell line

**Figure 4:**
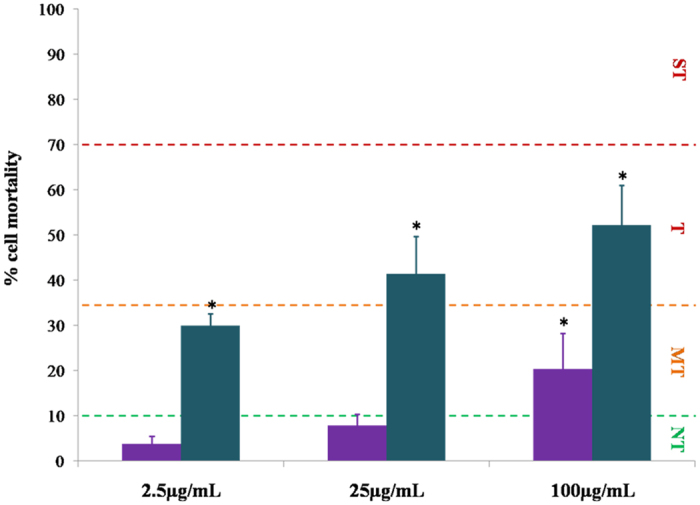
The % mortality of THP-1 cells upon exposure to various concentrations of aluminium adjuvants. Purple and green bars represent Alhydrogel^®^ and Adju-Phos^®^ respectively. The abbreviations ST, T, MT & NT represent the phrases severe toxicity, toxicity, moderate toxicity and null toxicity respectively. Error bars are representative of ±SD of 3 individual replicates and statistical significance is shown between treatments and respective control groups. Toxicity boundaries were adapted from Eidi *et al*.^24^.

The assessment of cellular toxicity was performed over 24 h using T helper 1 (THP-1) cells of a monocytic lineage. As a phagocytic cell line, THP-1 cells provide an excellent model to investigate phagocytosis of ABA, used in previous studies to assess adjuvanticity in cellular models of vaccination^16^,^18^,^25^. Singular treatments of Alhydrogel^®^ where concentrations of Al ≤ 25.0 μg/mL, did not facilitate a considerable reduction in cell viability following a 24 h exposure regime, as demonstrated by the low cellular mortality exhibited by these groups (Fig. 4) (<10.0%, P > 0.1 relative to the control group). However, a considerable elevation in cellular mortality was observed when the concentration of aluminium was increased to 100.0 μg/mL (20.3 ± 7.9%, P < 0.05). Alhydrogel^®^ was therefore found to be moderately cytotoxic to THP-1 cells.

By contrast, treatments of Adju-Phos^®^ induced a substantially greater level of cytotoxicity at lower concentrations of the adjuvant (Fig. 4). Cells treated with 2.5 μg/mL of the adjuvant induced a 29.9 ± 2.7% reduction in cell viability over a 24 h exposure period (P < 0.05 relative to the control). This level of cellular mortality was considered moderately cytotoxic and the abundance of non-viable monocytes continued to increase as the treatment dose was raised reaching *ca* 50.0% at 100.0 μg/mL. The level of cellular mortality, was hence considered to be above the moderate-toxicity threshold, above concentrations of 25.0 μg/mL of Adju-Phos^®^.

### The assessment of the cellular uptake of an aluminium oxyhydroxide-based adjuvant, via fluorescence microscopy

Autofluorescence analyses of THP-1 cells was assessed via analysis of 2 μm thick re-hydrated agar-paraffin cell sections in the absence of the fluor, lumogallion. No detectable autofluorescence signal was observed from THP-1 cells whether co-cultured in the absence or presence of an ABA. Native THP-1 cells cultured in the presence of R10 medium only and stained with lumogallion for 24 h, produced a dull orange/brown fluorescence under the lumogallion fluorescence channel (see Supplementary Fig. 2). Counter-staining with 4′,6-diamidino-2-phenylindole, dihydrochloride (DAPI), confirmed the presence of cells and specifically cell nuclei, as observed by a blue fluorescence emission. Merging of the fluorescence channels with the bright field image, revealed a dull orange/brown fluorescence contained exclusively within the cytosol of THP-1 cells. Extracellular fluorescence through either the lumogallion or DAPI fluorescence channel was not observed for native cells.

**Figure 5:**
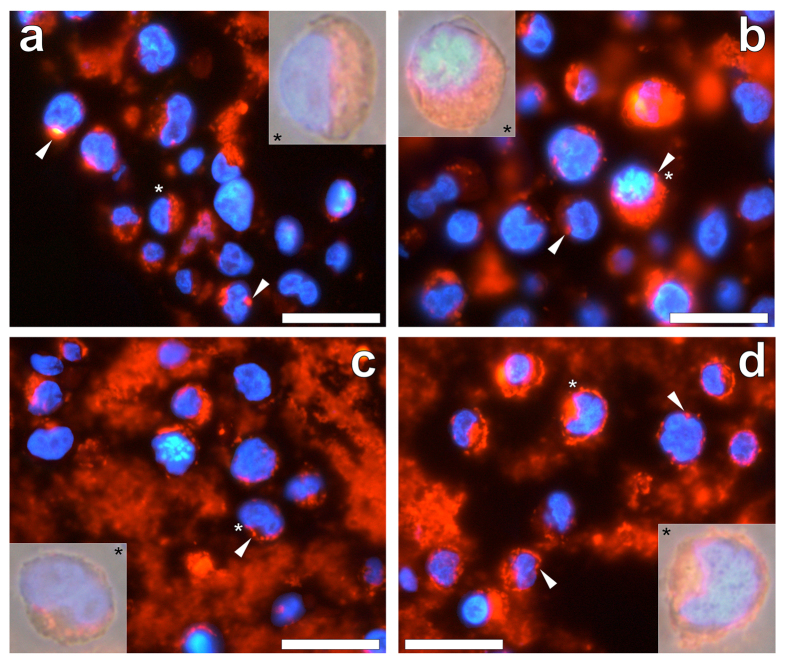
Representative lumogallion staining of agar-paraffin embedded (2 μm sections) THP-1 cells co-cultured with (**a**) 2.5, (**b**) 25.0, (**c**) 50.0 or (**d**) 100.0 μg/mL Alhydrogel^®^ (Brenntag Biosector, Denmark). Cell sections were incubated for 24 h in 100 μM lumogallion, 50 mM PIPES, pH 7.4. Slides were mounted with ProLong^®^ Gold Antifade Reagent with DAPI. All images depict lumogallion staining (orange) overlaid with DAPI-staining (blue). Magnified inserts show close-ups of individual cells with the light channel overlaid. White arrows highlight both individual and distinguishable adjuvant particles. Magnification X 1000, scale bars: 20 μm.

In order to investigate the potential cellular uptake of increasing concentrations of Alhydrogel^**®**^, THP-1 cells were co-cultured in the presence of 2.5, 25.0, 50.0 or 100.0 μg/mL of the adjuvant for 24 h (see Supplementary Fig. 3). THP-1 cells co-cultured in the presence of 2.5 μg/mL Alhydrogel^**®**^ and stained with lumogallion for 24 h, revealed bright punctuate fluorescence at their periphery (Fig. 5a). Overlaying of the DAPI fluorescence and light channels confirmed that lumogallion fluorescence of the ABA was contained solely within cell cytosol. THP-1 cells co-cultured with 25.0 μg/mL Alhydrogel^**®**^ produced clear orange fluorescence in the form of spherical particulate-like structures that were found to be heavily loaded in cytosolic compartments (Fig. 5b). In addition, all cells identified by DAPI fluorescence, confirmed the presence of internalised lumogallion-positive particulates. As with cells co-cultured in the presence of 2.5 μg/mL of the ABA, minimal traces of extracellular adjuvant material were observed.

For cells co-cultured in the presence of 50.0 μg/mL Alhydrogel^**®**^, merging of fluorescence and light channels revealed heavy loading of lumogallion-positive particulate-like structures within the minimal cytoplasmic space of THP-1 cells (Fig. 5c). Extracellular lumogallion fluorescence was observed for cells co-cultured in the presence of 50.0 and 100.0 μg/mL Alhydrogel^**®**^. Interestingly, cells co-cultured at the highest concentration of the ABA (Fig. 5d) revealed the greatest cytoplasmic loading of lumogallion fluorescent particulates. Furthermore, only at the highest concentration of the ABA were fluorescent particulates found adsorbed to the plasma membrane of THP-1 cells, as revealed by overlaying the bright field image (Fig. 5d). Minimal variation in the size of intracellular particulates of Alhydrogel^**®**^ was found upon co-culture with THP-1 cells (see Supplementary Table 2), of which measurements across all concentrations produced an average outer diameter of 0.96 ± 0.19 μm (mean ± SD, *n* = 340).

### Comparative assessment of the cellular uptake of an aluminium hydroxyphosphate-based adjuvant formulation

**Figure 6:**
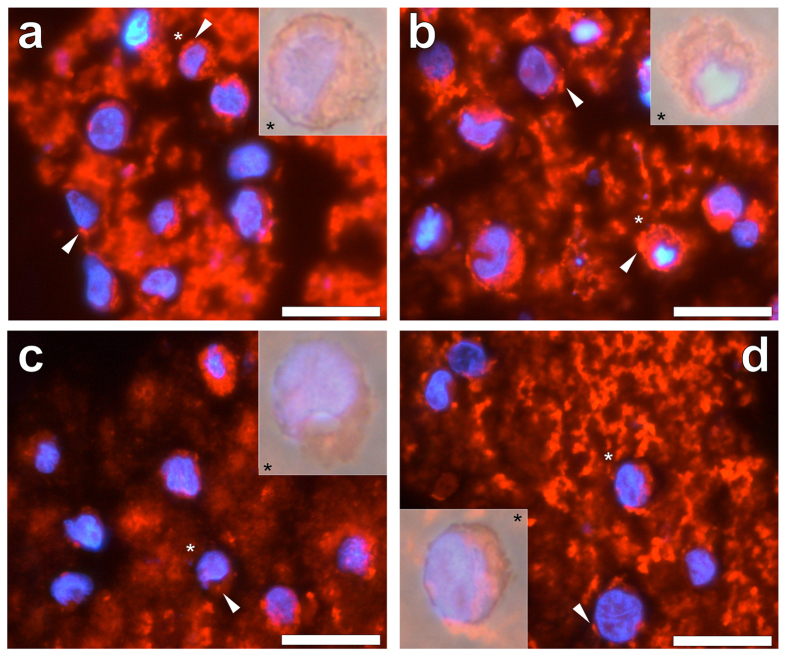
Representative lumogallion staining of agar-paraffin embedded (2 μm sections) THP-1 cells co-cultured with (**a**) 2.5, (**b**) 25, (**c**) 50 or (**d**) 100 μg/mL Adju-Phos^®^ (Brenntag Biosector, Denmark). Cell sections were incubated for 24 h in 100 μM lumogallion, 50 mM PIPES, pH 7.4. Slides were mounted with ProLong^®^ Gold Antifade Reagent with DAPI. All images depict lumogallion staining (orange) overlaid with DAPI-staining (blue). Magnified inserts show close-ups of individual cells with the light channel overlaid. White arrows highlight both individual and distinguishable adjuvant particles. Magnification X 1000, scale bars: 20 μm.

For cells co-cultured in the presence of 2.5–100.0 μg/mL of an amorphous aluminium hydroxyphosphate-based Adju-Phos^®^ adjuvant, a bright orange fluorescence was observed at the periphery of cells (see Supplementary Fig. 4). Merging of the lumogallion, DAPI and light channels revealed that particulates of the adjuvant were dispersed exclusively throughout the cell cytosol (Fig. 6). Adju-Phos^®^ particulates as highlighted by lumogallion fluorescence (Fig. 6), were found to be more difficult to distinguish in comparison to cells co-cultured with Alhydrogel^®^ (Fig. 5). Whilst the cellular internalisation of Adju-Phos^®^ within the cytoplasm of THP-1 cells was highlighted via lumogallion staining, the uptake of particulates was found to be less well pronounced at the highest concentrations of 50.0 and 100.0 μg/mL (Fig. 6d) of the adjuvant. Internalised particulates of Adju-Phos^®^ appeared larger than those of Alhydrogel^®^ and their size remained consistent across all concentrations of the ABA investigated, with an average outer diameter of 1.31 ± 0.22 μm (mean ± SD, *n* = 137) (see Supplementary Table 2).

Extracellular deposits of both amorphous and lumogallion-reactive Adju-Phos^®^ were readily identified at every adjuvant concentration investigated. Interestingly, counterstaining with the nucleic-reactive dye, DAPI, revealed that at concentrations as low as 2.5 μg/mL of Adju-Phos^®^, extracellular genetic material was observed (see Supplementary Fig. 5). No correlation could be drawn however, between the proportions of intact versus fragmented cell nuclei upon an increasing adjuvant concentration whereas cell nuclei were found to remain intact at all concentrations analysed for Alhydrogel^®^ (see Supplementary Fig. 3).

### Assessment of the cellular internalisation of an experimental aluminium hydroxycarbonate and magnesium hydroxide-based adjuvant.

**Figure 7:**
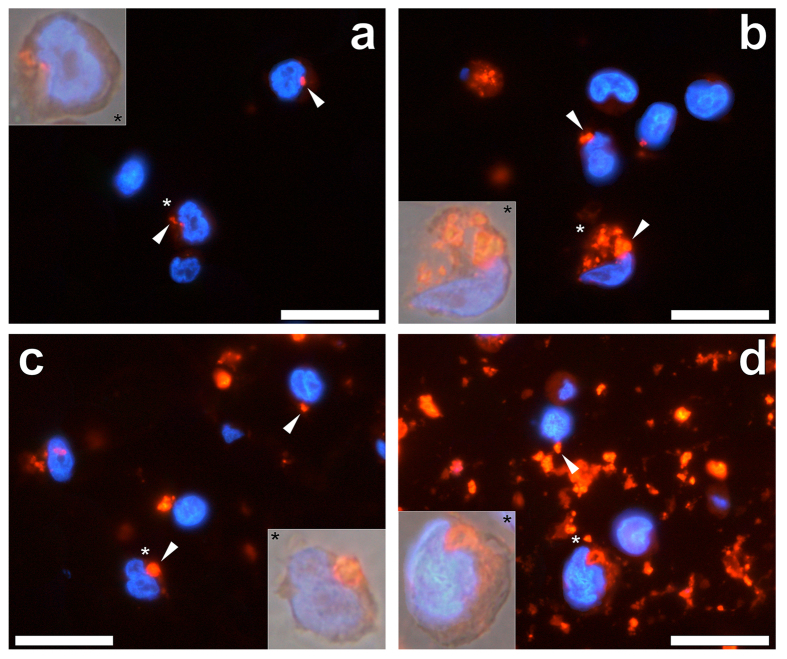
Representative lumogallion staining of agar-paraffin embedded (2 μm sections) THP-1 cells co-cultured with (**a**) 2.5, (**b**) 25, (**c**) 50 or (**d**) 100 μg/mL Imject™ Alum (Thermo Scientific). Cell sections were incubated for 24 h in 100 μM lumogallion, 50 mM PIPES, pH 7.4. Slides were mounted with ProLong^®^ Gold Antifade Reagent with DAPI. All images depict lumogallion staining (orange) overlaid with DAPI-staining (blue). Magnified inserts show close-ups of individual cells with the light channel overlaid. White arrows highlight both individual and distinguishable adjuvant particles. Magnification X 1000, scale bars: 20 μm.

The extracellular uptake of Imject™ Alum was found to be less pronounced at 2.5 μg/mL in comparison to the clinically used ABAs, as revealed through the lumogallion fluorescence channel (Fig. 7a). Those internalised particulates observed were also found to vary considerably in size with an average outer diameter of 2.11 ± 0.78 μm (mean ± SD, *n* = 17) (see Supplementary Table 2). Cellular internalisation of Imject™ Alum was also identified at 25.0 and 50.0 μg/mL, as identified by lumogallion fluorescence (Fig. 7b,c). The cellular uptake of Imject™ Alum was less pronounced at 100.0 μg/mL however, overlaying of the bright field image confirmed that those particulates internalised were confined within the cell cytoplasm of THP-1 cells (Fig. 7d). In spite of modifications in the agar-paraffin embedding protocol employed (see Supplementary methods), extracellular material remained identifiable via lumogallion fluorescence at 100.0 μg/mL of the ABA (see Supplementary Fig. 6). Extracellular genetic material was not observed at any adjuvant concentration analysed. Measurements of the size of internalised particles of Imject™ Alum were found to vary considerably from cell to cell under all treatment conditions with an average outer diameter of 1.90 ± 0.81 μm (mean ± SD, *n* = 93) (see Supplementary Table 2).

### Transmission electron microscopy reveals morphological variations of internalised aluminium based adjuvant particulates.

**Figure 8:**
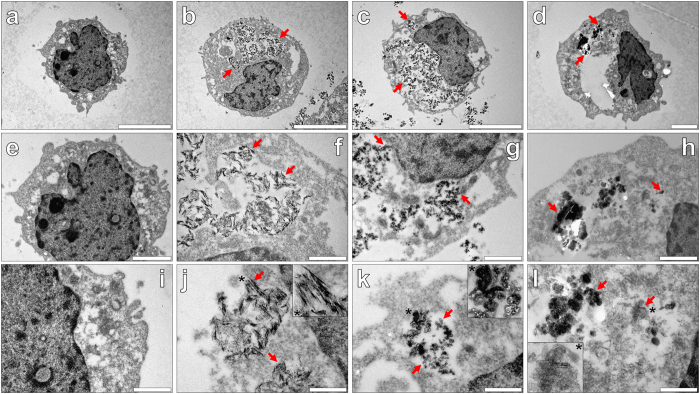
Representative electron micrographs from TEM of Spurr resin-sectioned (100 nm sections) native THP-1 cells (**a**,**e**,**i**), THP-1 cells co-cultured with 50 μg/mL Alhydrogel^®^ (24 h) (**b**,**f**,**j**) and 50 μg/mL Adju-Phos^®^ (Brenntag Biosector, Denmark) adjuvant (24 h) (**c**,**g**,**k**) and 50 μg/mL Imject™ Alum (Pierce, Thermo Scientific) adjuvant (24 h) (**d**,**h**,**l**). Cell resin-sections were stained for 20 min with 2% ethanolic uranyl acetate, rinsed with 30% ethanol followed by ultrapure water and finally allowed 24 h drying time prior to analysis via TEM. Inserts show close-ups of intracellular adjuvant particles contained within vesicle-like structures and the red arrows highlight their presence within the respective cell images. Magnification and scale bars: (**a–c**) X 8 K, 5 μm, (**d**) X 10 K, 2 μm, (**e)** X 15 K, 2 μm, (**f**–**h**) X 30 K, 1 μm, (**i**) X 30 K, 1 μm and (**j**–**l**) X 60 K, 0.5 μm, respectively.

In order to reveal potential morphological variations between internalised particulates of the differing ABA investigated, 50.0 μg/mL of each was co-cultured for 24 h with THP-1 cells and analysed by TEM^16^. Negative staining of native THP-1 cells cultured in R10 medium only, revealed the absence of internalised particulate material and a clear distinction between a granular cell cytosol and positively stained cell nuclei (Fig. 8a,e,i). TEM of cells co-cultured in the presence of Alhydrogel^®^ confirmed the internalisation of adjuvant particles (Fig. 5), of which electron dense particulates were found contained within the cytoplasm only (Fig. 8b). Higher magnifications revealed needle-like crystalline rods contained within clear cellular vesicles (Fig. 8f,j). Particle size measurements of internalised aggregates of Alhydrogel^®^ (see Supplementary Table 2) were found to closely correspond with those deposits detected via fluorescence microscopy, with an average outer diameter of 0.91 ± 0.14 μm (mean ± SD, *n* = 17). Owing to the needle-like morphology of those intracellular shaped crystals observed (Fig. 8j), their dimensions could be determined of which single crystals produced an average width of 8.2 ± 1.2 nm, of 54.1 ± 7.7 nm in length (mean ± SD, *n* = 17).

THP-1 cells co-cultured in the presence of Adju-Phos^®^ revealed positively stained amorphous aggregates in apparent cellular vesicles with an average size of 1.09 ± 0.25 μm (mean ± SD, *n* = 17) (see Supplementary Table 2) (Fig. 8c). Higher magnifications demonstrated that internalised aggregates of the adjuvant were constituted of negatively stained plate-like structures with an outer diameter of 15.0 ± 2.9 nm (mean ± SD, *n* = 17) (Fig. 8g,k). Intracellular particulates of Adju-Phos^®^ were found more greatly dispersed throughout the cytoplasm of THP-1 cells, than those co-cultured in the presence of Alhydrogel^®^. Furthermore, a loss in integrity of the cell cytoplasm for cells co-cultured in the presence of Adju-Phos^®^ was revealed, in comparison to cells co-cultured in the absence (Fig. 8a,e,i) or presence (Fig. 8b,f,j) of Alhydrogel^®^.

THP-1 cells co-cultured with 50.0 μg/mL Imject™ Alum contained large and amorphous particulates within cell cytosol only, with an average outer diameter of 1.21 ± 0.27 μm (mean ± SD, *n* = 3). Dense positive staining was observed for internalised particles of the adjuvant, with magnifications of X 30 K and higher revealing densely stacked and negatively stained plates, with an approximate outer diameter of 67.9 ± 10.8 nm (mean ± SD, *n* = 17). Minimal losses in the integrity of the cell cytoplasm was found, as for cells co-cultured in the absence or presence of Alhydrogel^®^. Higher magnifications of X 60 K, revealed a second needle-like crystalline morphology of internalised particulates of Imject™ Alum within the cytoplasm of THP-1 cells (Fig. 8l). The electron micrographs obtained therefore depicted two distinct particulate morphologies, which suggested their co-existence in the ABA formulation, or their potential degradation in the cytosolic compartment of THP-1 cells.

## Discussion

We have drawn the direct comparative assessment of the physicochemical properties and biological factors underlying the unequivocal cellular uptake and adjuvanticity of clinically relevant and experimental ABA formulations, in a relevant THP-1 cell model of vaccination^16^,^18^,^25^. Herein, we have demonstrated that of the most commonly used ABA in clinically approved vaccinations, neither aluminium oxyhydroxide (Alhydrogel^®^) nor aluminium hydroxyphosphate (Adju-Phos^®^) are equivalent in their physico-chemistry nor in their biological reactivity. Our results continue to raise concern^26^ over use of the experimental and chemically different, aluminium hydroxycarbonate and magnesium hydroxide based Imject™ Alum formulation^27^, as the model adjuvant of choice in the study of clinical vaccination. Furthermore Imject™ Alum has been shown to elicit weaker humoral T_H_2 immune responses via diminished IgG antibody production and reduced pro-inflammatory cytokine release versus Alhydrogel^®^, in a nitrophenol-chicken-gamma-globulin hapten carrier antigen (NP-CGG) model of murine vaccination^26^.

Striking differences in separate reports of the immunomodulatory properties of ABA have been linked to variations in adjuvant formulation and their respective dose^3^,^10^. For example, the important discovery of the multiprotein Nalp3 inflammasome^22^ is now regarded as playing a contributory^3^ rather than a crucial role in the adjuvanticity of ABA through driving inflammation through IL-1β and IL-18 release^22^,^28^. As such, our initial efforts were focused upon establishing the physicochemical properties of the clinically relevant Alhydrogel^®^ and Adju-Phos^®^ adjuvants, with respect to their particle size and surface zeta potential or charge, in a proteinaceous R10 cell culture medium, modelling MIF at the injection site^29^,^30^.

It was observed that whilst Alhydrogel^®^ was found positively charged conversely to negatively charged Adju-Phos^®^ in simulated 0.9% NaCl vaccine diluent, once introduced into R10, both formulations possessed a steady net negative charge. Complementary analyses via TEM at 0 h confirmed the rapid formation of an extensive protein corona in R10 formed around particulates of each ABA thereby explaining the masked and equivalent surface charges *in vitro* (Fig. 3). Hence the rapid dissolution of the antigen from its ABA upon competing interactions with interstitial proteins^31^, may explain reported discrepancies in the depot effect, via gradual and sustained antigen released^10^.

Particle sizes of Alhydrogel^®^ in R10 by DLS showed the gradual aggregation of adjuvant, with particles present in the micron range of *ca* 1.4 μm, following 24 h, in contrast to Adju-Phos^®^ of which particles were observed to disaggregate from 2.2 to *ca* 1.8 μm (Fig. 1). Due to the rapid molecular process of phagocytosis in which particles are typically engulfed in fewer than twenty minutes^32^, Alhydrogel^®^ is more predisposed to lie in the optimal size range of phagocytosis from 0.5–5.0 μm^33^, in contrast to Adju-Phos^®^ over the first 24 h. The latter is most likely only internalised by phagocytic entry at the earliest time points as approximately 80% of particulates lie outside of phagocytic range by 1 h, increasing to 97% by 24 h.

Monocytes are typically recruited to the injection site following 24 h from initial administration of an ABA^34^ and subsequently dominate phagocytic clearance of particulates over early infiltrating neutrophils^35^, in the size range of 0.5–5 μm^33^. Taken collectively, our results therefore predict that Adju-Phos^®^ is most likely phagocytosed by neutrophils over Alhydrogel^®^ adjuvanted vaccines, whereby phagocytic clearance is governed by monocytes at the injection site. The enhanced phagocytosis of Adju-Phos^®^ by infiltrating neutrophils over the earliest time points following vaccination may only be speculated at present, in the absence of *in vitro* data demonstrating such unequivocal cellular uptake.

Whilst the infiltration of macrophages at the injection site seven days post-vaccination would likely govern the phagocytic clearance of particulate ABA immune agonists at the injection site^4^,^17^,^34^, all aforementioned cell types may act as antigen presenting cells (APCs)^36^, capable of triggering adaptive immune responses^10^. Therefore we next addressed the unequivocal cellular uptake of ABA using lumogallion as an established fluorescent molecular probe for the detection of intracellular aluminium^16^,^17^,^25^,^37^.

Regardless of the ABA investigated intracellular adjuvant particulates were only found localised in the cytoplasm of THP-1 cells. Alhydrogel^®^ was found internalised at every concentration investigated (Fig. 5), whereas for both Adju-Phos^®^ (Fig. 6) and Imject™ Alum (Fig. 7), internalisation was less pronounced at concentrations at or exceeding 50.0 μg/mL of the respective adjuvant. At ABA concentrations where the cellular internalisation of particulates was always observed (2.5 to 25.0 μg/mL), Alhydrogel^®^ exhibited the greatest cytoplasmic particulate loading. Furthermore Alhydrogel^®^ was the only ABA investigated that was found co-adsorbed to the plasma membrane of cells where near complete loading of the cytoplasm was observed at 100.0 μg/mL of the adjuvant. Intracellular ABA particulates across all ABA concentrations investigated, revealed that particulates of Imject™ Alum were greatest and most variable in size followed by Adju-Phos^®^ and finally Alhydrogel^®^ at *ca* 1.9, 1.3 and 1.0 μm, respectively (see Supplementary Table 2).

Intracellular particulates of Adju-Phos^®^ were found to be most heterogeneous, in which the diffuse lumogallion staining observed was likely a result of the ease of solubilisation of the amorphous adjuvant. Strikingly, only cells co-cultured with Adju-Phos^®^ were found to release genetic material into the extracellular milieu at concentrations as low as 2.5 μg/mL of the adjuvant (see Supplementary Fig. 5). Adju-Phos^®^ was also found more cytotoxic to THP-1 cells than Alhydrogel^®^, of which less than 50% of cells were found viable at the highest concentration of the adjuvant analysed at 100.0 μg/mL (Fig. 4). In contrast, Alhydrogel^®^ was only found moderately cytotoxic to cells above the control group at 100.0 μg/mL. The extracellular release of DNA from dying cells has been found to enhance the adjuvanticity of ABA as DAMPs that activate the Nalp3 inflammasome^2^,^38^. Therefore, the reduced cellular uptake of amorphous Adju-Phos^®^ and its enhanced cytotoxicity at the injection site speculatively through Al^3+^_(aq)_ release^4^,^17^, in comparison to semi-crystalline Alhydrogel^®^ 8,27, may act to mediate its adjuvanticity by an alternative cellular pathway of immunopotentiation.

Finally TEM of cells co-cultured with ABA was performed in order to uncover the potential morphological variations of those intracellular particulates revealed by fluorescence microscopy. TEM revealed the same trend in intracellular ABA particulate sizes (Imj > Adj > Alh) with smaller particle sizes attributed to thinner sectioning at 100 nm necessary for TEM^16^. As with fluorescence microscopy, particulates of ABA were solely found contained within the cytoplasm only of THP-1 cells (Fig. 8). Semi-crystalline rods of Alhydrogel^®^ of length and width 54.1 ± 7.7 and 8.2 ± 1.2 nm (mean ± SD, *n* = 17) respectively, were found readily internalised within vesicular-like structures within THP-1 cells. Measurements of intracellular partially ordered needles of Alhydrogel^®^ were found to constitute aggregates of primary particles of 4.5 × 2.2 × 10.0 nm known for structurally related boehmite preparations as confirmed by the complementary use of X-ray crystallography and Fourier transform infrared spectroscopy, respectively^8^.

Intracellular and individual plate-like structures of Adju-Phos^®^ were found surprisingly smaller at 15.0 ± 2.9 nm than single needles of Alhydrogel^®^, that whilst contained in endosomal compartments, lacked membrane enclosure. In support of those findings from fluorescence microscopy, the smaller particle sizes observed demonstrated the ease of dissolution of Adju-Phos^®^ in acidified cellular compartments and as such may also explain greater distribution of amorphous particulates of the adjuvant throughout THP-1 cell cytosol. Furthermore, a loss of integrity of the plasma membrane was observed for cells co-cultured with Adju-Phos^®^ thereby explaining the release of genetic material^38^ through cytotoxicity and potential cellular lysis. Comparative analyses of the experimental Imject™ Alum formulation via TEM revealed the largest of all single intracellular ABA particulates of 67.9 ± 10.8 nm, in which negatively stained plates were observed stacked densely into amorphous aggregates. Needle-like crystals were also observed of crystalline magnesium hydroxide or speculatively additional breakdown products of the adjuvant.

Overall TEM provided insight for the first time of the capability of THP-1 cells to internalise multiple ABA morphologies most notably through phagocytosis resulting in their presence in autolysosomal vesicles^16^,^17^,^18^,^25^ as was especially prevalent for Alhydrogel^®^. In spite of Imject™ Alum demonstrating the largest and most heterogeneous of intracellular ABA particulates of those formulations investigated, no loss in the cytoplasmic integrity was observed unlike those cells co-cultured in the presence of Adju-Phos^®^. Phagocytosis is regulated through reorganisation of the actin cytoskeleton and is necessary to allow for the cellular uptake of particulate material^39^. This mechanism of cellular uptake is known to be of detriment to the cell however, due to formation of large pores in the plasma membrane^39^. Taken collectively our results thereby support that the release of extracellular DNA and the cytotoxicity of Adju-Phos^®^ may be additionally governed by the release of Al^3+^_(aq)_ at the injection site^4^,^17^.

In conclusion, our results demonstrate through minimal cytotoxicity and high cytoplasmic loading that Alhydrogel^®^ as the most commonly used ABA in clinically approved vaccinations is most pre-disposed to migration away from the injection site through migratory phagocytic cell lineages. It is known that monocytes are capable of differentiating into either macrophagic or dendritic cell types^36^ and both have subsequently been linked to the presence of increased MHCII-positive DCs at the injection site, seven days following vaccination^34^,^35^. As such, migratory APCs including monocytes containing the internalised antigen may enter lymph nodes via draining through high endothelial venules (HEVs)^36^.

Bone marrow derived dendritic cells (BMDCs), have been previously utilised to further understanding of the biological mechanisms of action of ABA^21^. As professional antigen APCs representing the innate arm of the immune system, DCs are pivotal in driving an adaptive immune response in vaccination^10^. ABA have been suggested to promote an abortive non-endocytic pathway of the antigen only across DC membranes through lipid rearrangement^40^. Therefore, BMDCs are not predicted to phagocytose ABA. Regardless of whether ABA are phagocytosed by BMDCs or other APCs, the migration of ABA loaded monocytes to sites distant to the injection site, would establish their enhanced transport to resting DCs. Through subsequent activation of the Nalp3 inflammasome^10^,^22^ and enhanced MHCII-presented antigen site expression upon the surface of activated DCs^21^, an enhanced engagement of naïve CD4^+^ T cells takes place. The subsequent stimulation of T-cells through antigen presentation to resident B-cells, triggers plasma cell formation and the production of antibodies through T_H_2 humoral immune responses, effectively combatting the target antigen^2^,^10^,^23^,^36^.

Through *in vitro* cellular modelling, our results further shed light on the capacity of ABA to deposit at sites distant to the injection site as has been suggested in macrophagic myofasciitis (MMF), whereby aluminium is proposed to translocate through draining lymph nodes to distant organs^24^,^41^.

## Methods

### Dynamic light scattering and zeta potential analysis

Particle size and zeta potential analysis was performed using a Zetasizer Nano ZS (Malvern Instruments, UK) equipped with a red laser (633 nm) whose detection ranges for each method were 0.6 nm–6.0 μm and 3.0 nm–10 μm respectively. Briefly, vaccine preparations were introduced into R10 medium at 37 °C and analysed after incubation periods of 0, 1 and 24 h. The final concentration of aluminium within the R10 medium post vaccine administration was *ca* 130 μg/mL. A total of five measurements were made per sample replicate (*n* = 5) and the average of these was used to derive size distribution and zeta potential values.

### Size exclusion filtration of aluminium adjuvants

Size exclusion of samples was performed using syringe filtration through a variety of membranes, which were sterilised via exposure to ethylene oxide or γ radiation prior to purchase (see Supplementary Table 3). PTFE membranes were treated with 70% *v/v* ethanol prior to use to achieve a hydrophilic surface suitable for aqueous filtration. Membrane affinity for serum proteins and aluminium was evaluated as part of method optimisation. Briefly, before each filtration step samples were inverted briefly in an attempt to equivalently distribute particulates. Aluminium adjuvant treatments were sequentially filtered through 5.6, 2.7 and 0.25 μm, PVDF, PTFE and PVS membranes respectively, in order to prevent unnecessary blockage of filter membranes with smaller nominal pore sizes. Filtrates were retained for the analyses set out herein.

### Graphite furnace atomic absorption spectroscopy of aluminium adjuvant filtrates

The protocol used for the quantification of aluminium within filtrates was adapted from an established method by House *et al*.^42^. Briefly experimental solutions and corresponding filtrates (1 mL) were acidified to 50% *v/v* using concentrated HNO_3_ (15.8 M, analytical grade - Fisher scientific, UK). Acidified solutions, which included negative nitric acid controls, were digested using a 1800 watt MARS 6 Xpress microwave digester (CEM Corp., US) at 180 °C for 40 mins. Quantification of solution aluminium content was performed using an AAnalyst 600 atomic absorption spectrometer complete with transversely heated graphite atomizer (THGA) and AS 800 autosampler (Perkin Elmer, UK). The equipment was calibrated using freshly prepared aluminium solutions in 1% HNO_3_ (max. 60 μg/L Al) and digests were diluted in ultrapure water where appropriate prior to analysis.

### THP-1 cell culture and fixation

THP-1 cells (ATCC TIB-202, LGC Standards, UK) were cultured according to the manufacturer instructions as described in the supplementary methods online. Native THP-1 cells (i.e. those containing no ABA) were cultured in the presence of complete R10 medium only. Those cell treatments containing the ABA were co-cultured in R10 media containing a final concentration 2.5, 25.0, 50.0 or 100.0 μg/mL of either Alhydrogel^®^ (2%), Adju-Phos^®^ (both from Brenntag Biosector, Denmark) or Imject™ Alum (Thermo Scientific, Pierce) for 24 h. Cell treatments for fluorescence microscopy were prepared in 96 well plates using *ca* 3 × 10^5^ cells for each treatment condition, with a final volume of 200 μL per well. Following 24 h incubation, cells were aspirated from the wells and 10.0 mL of 0.22 μm filtered isotonic PIPES buffered saline (150 mM NaCl, 50 mM PIPES, pH 7.4) was added to each respective pooled cell treatment. Cell treatments were subsequently centrifuged at 800 *g* at ambient temperature for 10 min and the supernatants thereafter removed.

Cells were fixed by the addition of 0.22 μm filtered 4% *w/v* paraformaldehyde (4% *w/v* PFA, 150 mM NaCl, 25 mM PIPES, pH 7.4), proceeded by incubation at ambient temperature for 20 min. Fixed cells were subsequently pelleted and washed three times in isotonic PIPES buffered saline, prior to pre-embedding in an agar support medium.

### Cytotoxicity assay (Live/dead staining)

Live/dead staining kits purchased from Life technologies, UK were used to measure the viability of THP-1 cells, following treatment with aluminium adjuvants. Cells from parent cultures were harvested, counted and seeded into 96 well plates at a final density of *ca* 1 × 10^6^ cells/mL. Cells were treated with vaccine preparations containing 2.5, 25.0 and 100.0 μg/mL Al and subsequently incubated at 37 °C under humidified CO_2_ conditions (5%) for 24 h. Control and treated cells were isolated from the culture medium via centrifugation at 300 *g* for 10 mins and washed three times in 1X phosphate buffered saline (PBS) to reduce any residual esterase activity. Cells were re-suspended in fresh PBS and applied to a non-sterile black 96 well fluorescence plate (100 μL per well). The fluorescence intensity from each well was measured using a GloMax^®^-Multi+ microplate multimode reader (Promega, UK), as described in the supplementary methods.

### Lumogallion staining of cell sections

Rehydrated agar-cell sections were stained by fully immersing slides in 250.0 mL 100 μM lumogallion (TCI Europe N.V. Belgium) buffered in 50 mM PIPES, pH 7.4. For autofluorescence analyses of THP-1 cells, sections were placed into the same PIPES-buffer in the absence of added lumogallion. All sections were covered and incubated at ambient temperature in the dark for 24 h. Lumogallion stained cell sections were subsequently rinsed by agitation in 50 mM PIPES, pH 7.4 for 2 min, prior to rinsing for 30 s in ultrapure water to remove excess buffer. Sections for autofluorescence were rinsed in ultrapure water only. The latter was performed to prevent precipitation of PIPES upon the drying of cell sections. Sections were finally air dried and mounted using ProLong^®^ Gold Antifade Reagent with 4′,6-diamidino-2-phenylindole, dihydrochloride (DAPI) (Life Technologies, UK) prior to storing horizontally for 24 h at 4 °C.

### Fluorescence microscopy

DAPI-mounted THP-1 cell sections were viewed by use of an Olympus BX50 fluorescence microscope (mercury source). Fluorescence micrographs were obtained at X 1000 magnification using a X 100 Plan-Fluorite oil immersion objective (Olympus, UK) using a low auto-fluorescence immersion oil (Olympus immersion oil type-F). Fixed light transmission values were maintained across all respective treatment conditions and images obtained, using the Cell^D^ software (Olympus, Soft Imaging Solutions, GmbH) package. An Olympus U-MWU2 fluorescence filter cube (excitation: 300–385 nm, dichromatic: 400 nm, longpass emission filter: 420 nm) was used to assess DAPI fluorescence and for lumogallion imaging a single band pass emission filter (Chroma^®^, Vermont, US) was swapped into an existing U-MNIB3 fluorescence filter cube (excitation: 470–495 nm, dichromatic mirror: 505 nm, single band pass filter: 570–630 nm). Fluorescence and light channels were overlaid by the use of Photoshop (Adobe systems Inc. USA).

### Transmission electron microscopy (TEM)

THP-1 cell blocks were embedded into Spurr-resin as previously described^16^ and detailed in the supplementary methods. Spurr-resin embedded agar cell blocks were sectioned at 100 nm and floated onto ultrapure water. Sections were expanded using chloroform vapour and caught on 200 mesh, thin bar 3.05 mm copper grids (Athene, UK). Following 24 h, grids were negatively stained with 2% *w/v* uranyl acetate (UA) in 70% *v/v* ethanol. Briefly, grids were negatively stained in UA for 20 min, wicked and dipped 20 times in a large volume of 30% *v/v* ethanol. Grids were re-wicked after this step and rinsed by successively dipping 10 times in two changes of ultrapure water, with wicking performed between rinses. Grids were subsequently covered and allowed 24 h drying time, prior to analysis via TEM using a JEOL 1230 transmission electron microscope as described in the supplementary methods.

## Additional Information

**How to cite this article**: Mold, M. *et al*. Insight into the cellular fate and toxicity of aluminium adjuvants used in clinically approved human vaccinations. *Sci. Rep.* **6**, 31578; doi: 10.1038/srep31578 (2016).

## Supplementary Material

Supplementary Information
