# Supplementary material for: Insight into the cellular fate and toxicity of aluminium adjuvants used in clinically approved human vaccinations
Source: Sci Rep. 2016 Aug 12;6:31578. doi: 10.1038/srep31578 (PMC4981857; doi:10.1038/srep31578)
Supplement: Supplementary Information [file srep31578-s1.pdf]

Supplementary information

Title:

Insight into the cellular fate and toxicity of aluminium adjuvants used in clinically approved human vaccinations.

Author List:

Dr Matthew J. Mold: m.j.mold@keele.ac.uk

Dr Emma M. Shardlow: e.shardlow@keele.ac.uk

Prof Christopher Exley*: c.exley@keele.ac.uk

*****Corresponding author*

Postal address:

The Birchall Centre, Lennard-Jones Laboratories, Keele University, Keele, Staffordshire, ST5 5BG, UK.

Supplementary Figures

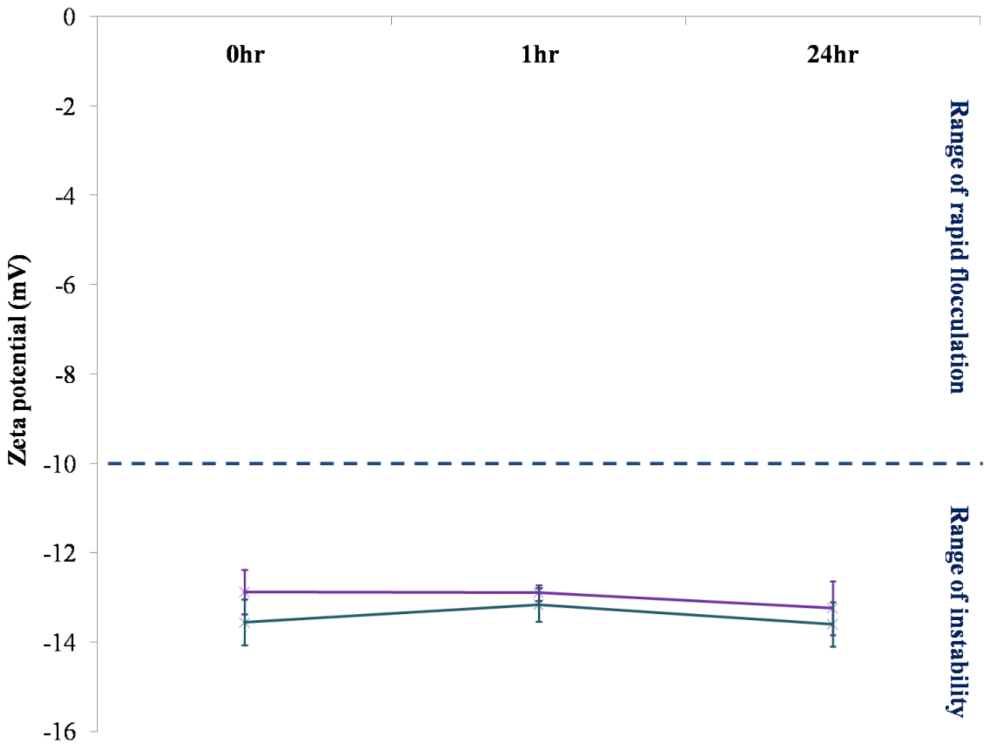

Supplementary Figure 1 Zeta potential characterisation of aluminium adjuvants following 0, 1 & 24 h incubation in R10 medium (37°C). Purple & green lines are representative of Alhydrogel® & Adju-Phos® respectively. Error bars represent the ±SD of the measurement where *n* = 5.

Fluorescence microscopy of native THP-1 cells cultured in the presence of R10 culture medium only and stained with lumogallion.

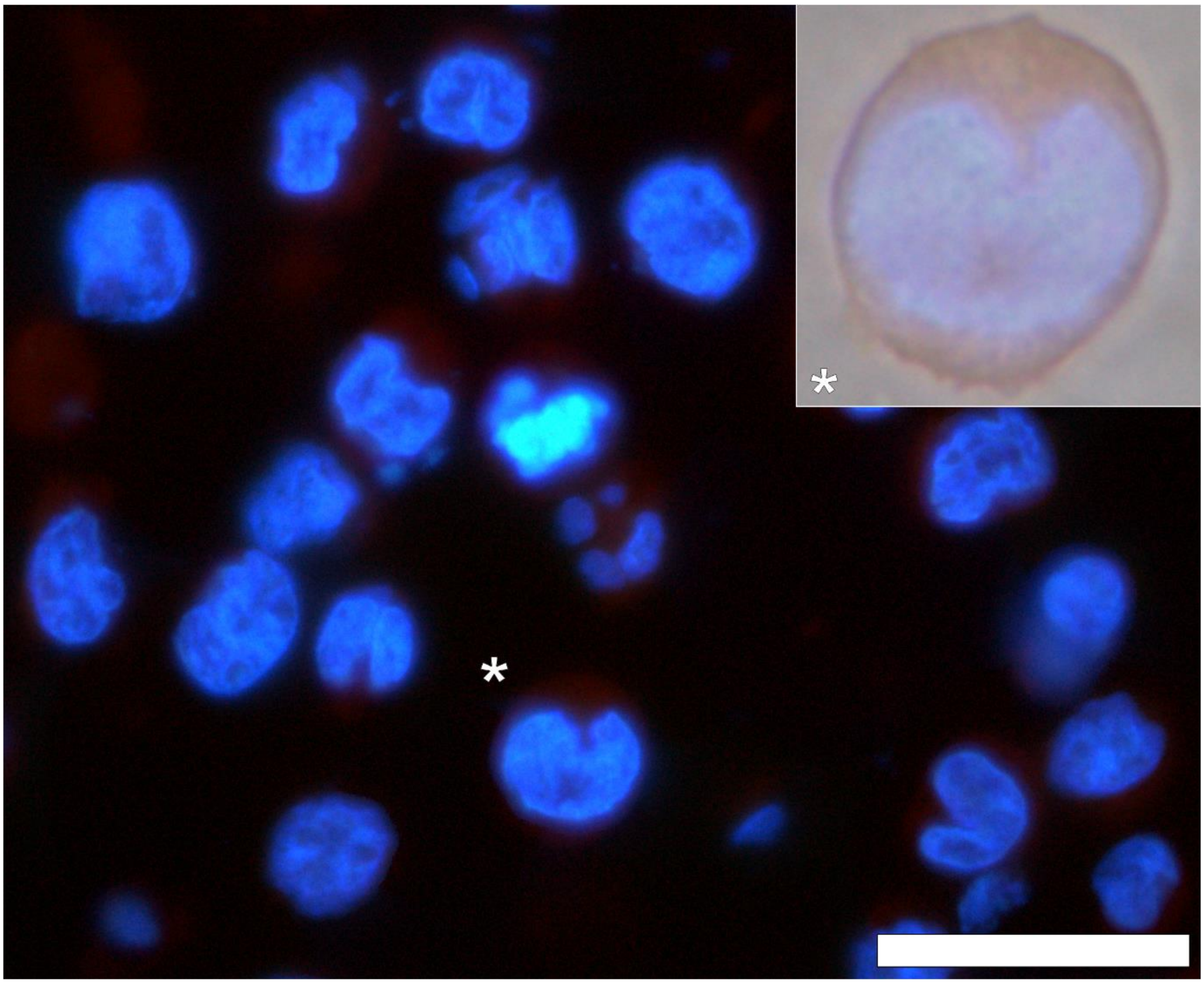

Supplementary Figure 2 Representative lumogallion staining of agar-paraffin embedded (2 µm sections) native THP-1 cells. Cell sections were incubated for 24 h in 100 µM lumogallion, 50 mM PIPES, pH 7.4. Slides were mounted with ProLong® Gold Antifade Reagent with DAPI. Lumogallion staining (orange) overlaid with DAPI-staining (blue). The magnified insert shows a close-up of an individual cell with the light channel overlaid. Magnification X 1000, scale bar: 20 µm.

Individual fluorescence channels used to identify increasing doses of an aluminium oxyhydroxide Alhydrogel® adjuvant in THP-1 cells.

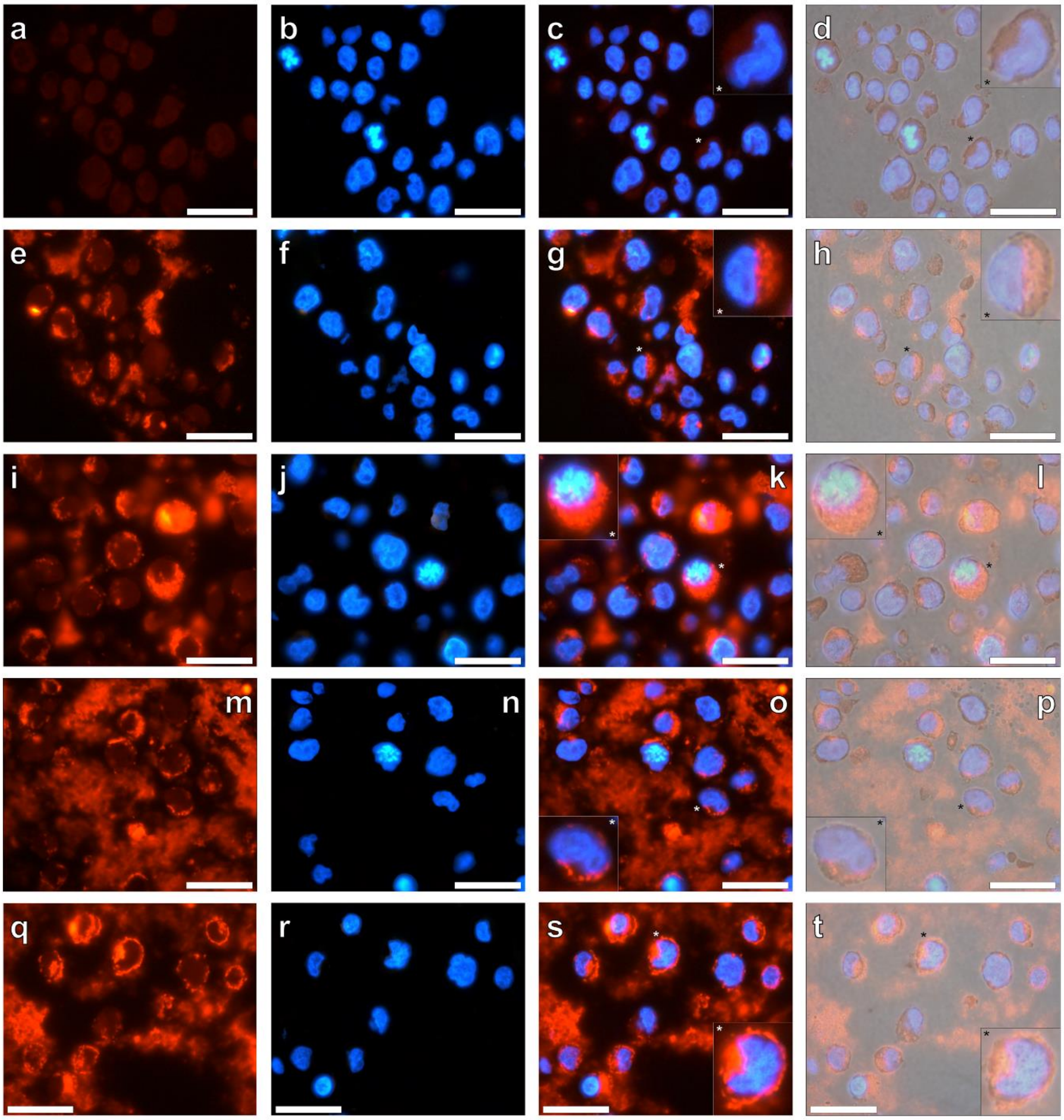

Supplementary Figure 3 Representative lumogallion staining of agar-paraffin embedded (2µm sections) THP-1 cells co-cultured in the absence (**a** – **d**) or presence of (**e** – **h**) 2.5, (**i** – **l**) 25, (**m** – **p**) 50 or (**q** – **t**) 100µg/mL Alhydrogel® (Brenntag Biosector, Denmark) for 24 h in 0.9% NaCl. Cell sections were incubated for 24 h in 100µM lumogallion in 50mM PIPES, pH 7.4. Sections were mounted with ProLong® Gold Antifade Reagent with DAPI. (**a, e, i, m, q**). lumogallion staining (orange), (**b, f, j, n, r**). DAPI-staining (blue), (**c, g, k, o, s**). lumogallion & DAPI overlay, (**d, h, l, p, t**). lumogallion, DAPI & light overlay. Inserts (marked by an asterisk) show close-ups of individual cells. Magnification X 1000, all scale bars: 20 µm.

Individual fluorescence channels used to identify increasing doses of an aluminium hydroxyphosphate Adju-Phos® adjuvant in THP-1 cells.

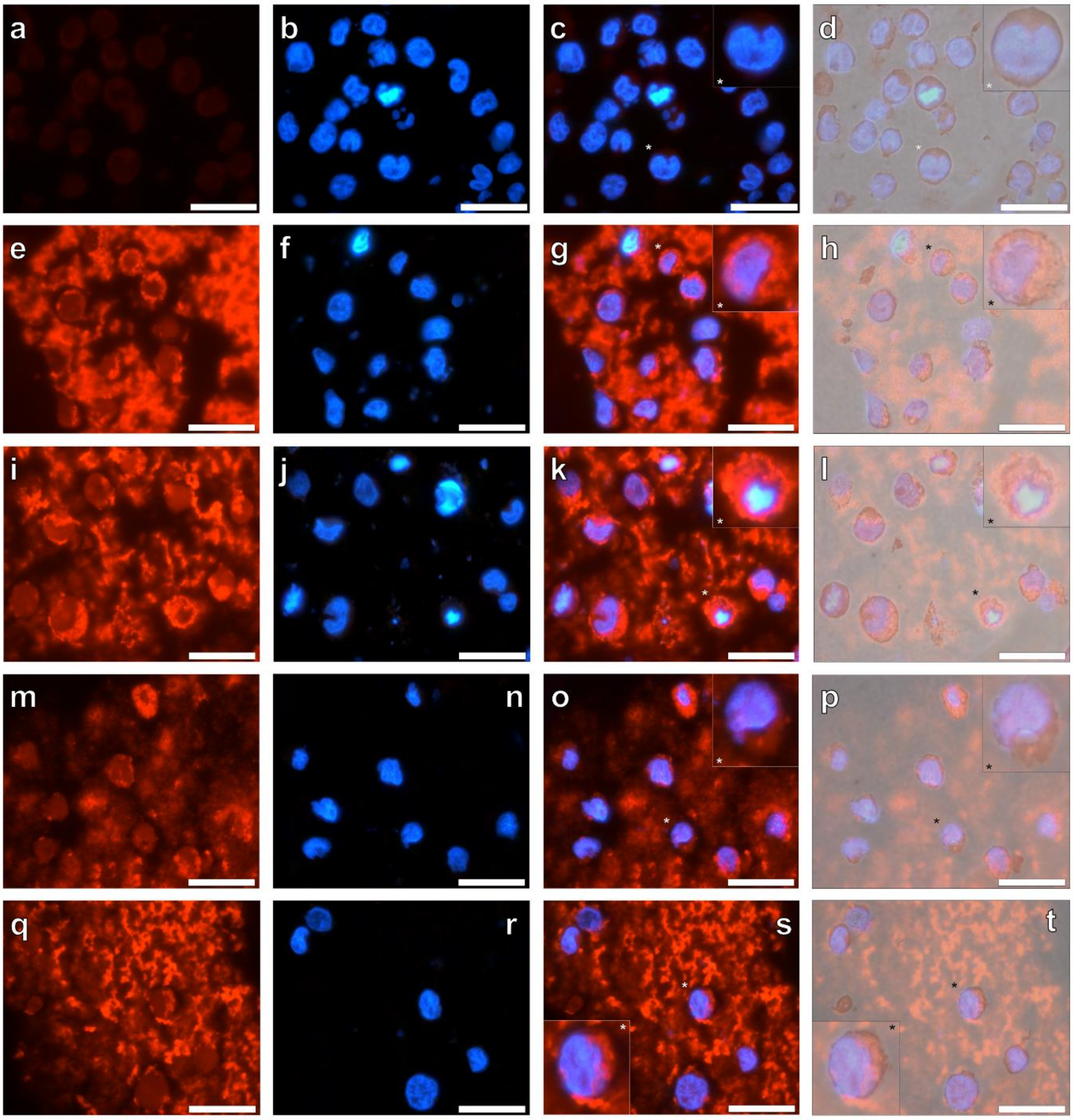

Supplementary Figure 4 Representative lumogallion staining of agar-paraffin embedded (2 µm sections) THP-1 cells co-cultured in the absence (**a – d**) or presence of (**e – h**) 2.5, (**i – l**) 25, (**m – p**) 50 or (**q – t**) 100µg/mL Adju-Phos® (Brenntag Biosector, Denmark) for 24 h in 0.9% NaCl. Cell sections were incubated for 24 h in 100µM lumogallion in 50mM PIPES, pH 7.4. Sections were mounted with ProLong® Gold Antifade Reagent with DAPI. (**a, e, i, m, q**). lumogallion staining (orange), (**b, f, j, n, r**). DAPI-staining (blue), (**c, g, k, o, s**). lumogallion & DAPI overlay, (**d, h, l, p, t**) lumogallion, DAPI & light overlay. Inserts (marked by an asterisk) show close-ups of individual cells. Magnification X 1000, all scale bars: 20 µm.

DAPI-fluorescence reveals extracellular genetic material from THP-1 cells in the presence of Adju-Phos®

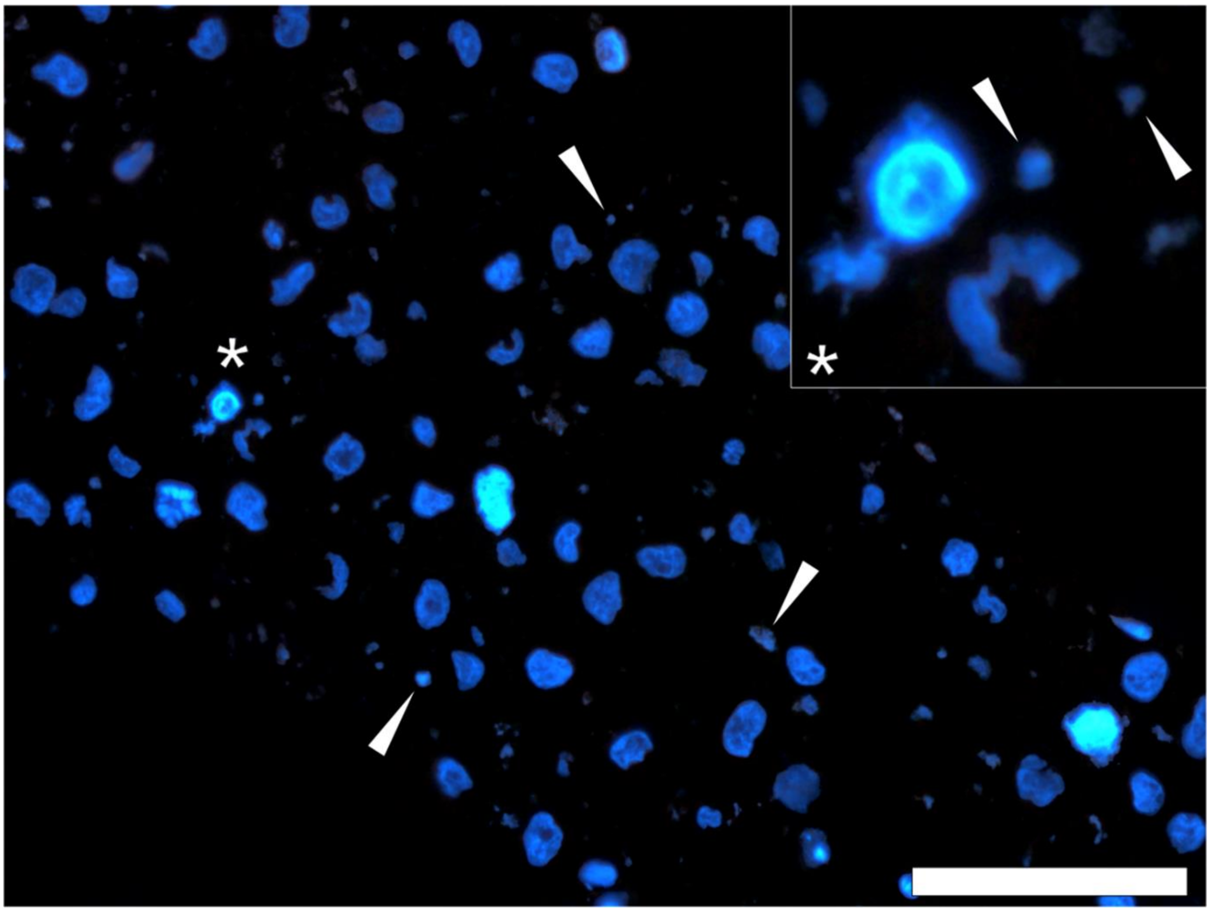

Supplementary Figure 5 Representative DAPI staining of agar-paraffin embedded (2 µm sections) THP-1 cells co-cultured in the presence of 2.5 µg/mL Adju-Phos® (Brenntag Biosector, Denmark). Cell sections were incubated for 24 h in 100 µM lumogallion, 50 mM PIPES, pH 7.4. Slides were mounted with ProLong® Gold Antifade Reagent with DAPI. DAPI-staining is depicted by a blue fluorescence emission. White arrows indicate extracellular genetic material and the magnified insert shows a close-up of DAPI-stained cell nuclei. Magnification X 400, scale bar: 50 µm.

Individual fluorescence channels used to identify increasing doses of an aluminium hydroxycarbonate, magnesium hydroxide Imject™ Alum adjuvant in THP-1 cells.

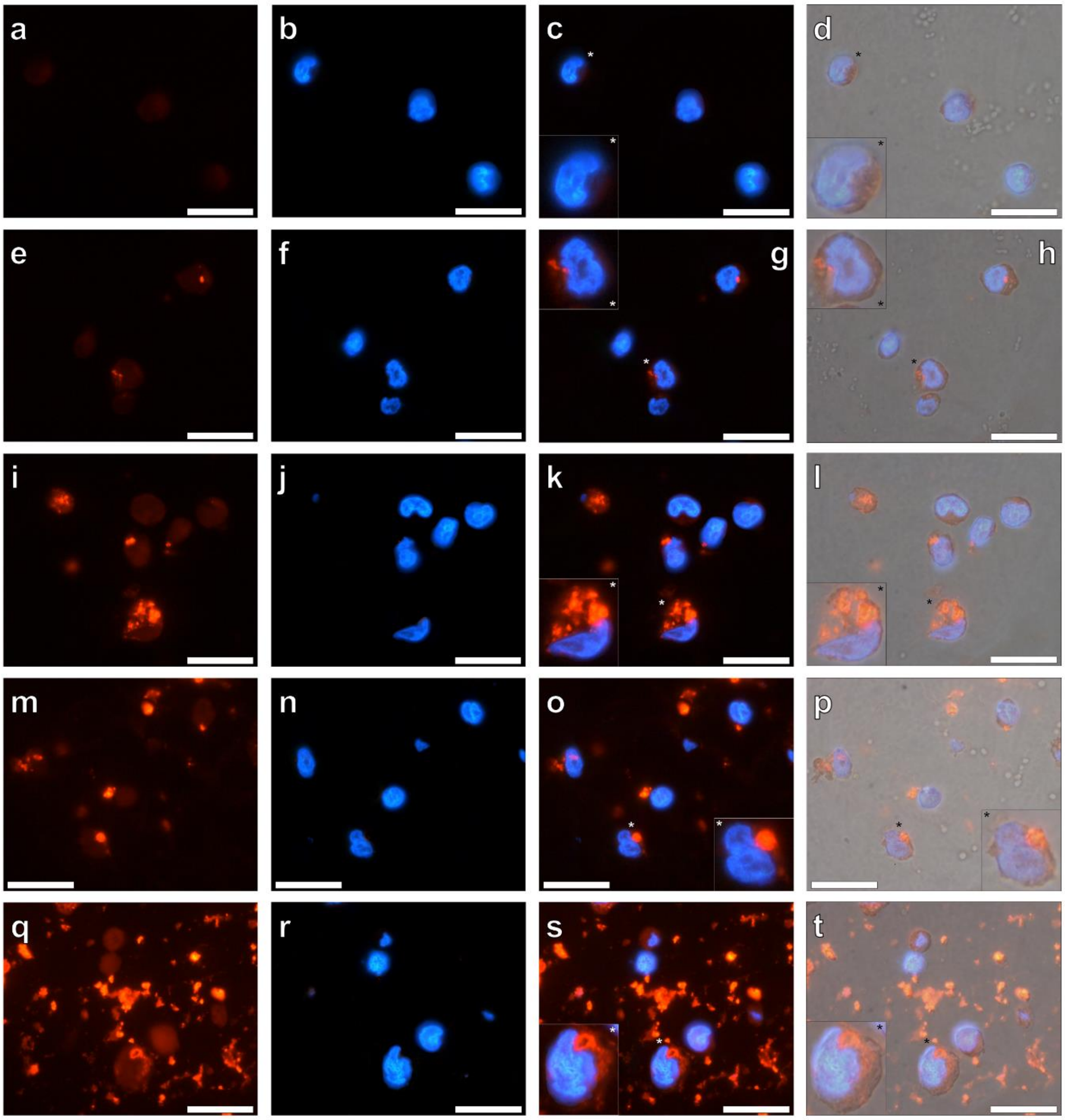

Supplementary Figure 6 Representative lumogallion staining of agar-paraffin embedded (2μm sections) THP-1 cells co-cultured in the absence of (**a** – **d**) or presence of (**e** – **h**) 2.5, (**i** – **l**) 25, (**m** – **p**) 50 or (**q** – **t**) 100μg/mL Imject™ Alum (Thermo Scientific) for 24 h in 0.9% NaCl. Cell sections were incubated for 24 h in 100μM lumogallion in 50mM PIPES, pH 7.4. Sections were mounted with ProLong® Gold Antifade Reagent with DAPI. (**a, e, i, m, q**). lumogallion staining (orange), (**b, f, j, n, r**). DAPI-staining (blue), (**c, g, k, o, s**). lumogallion & DAPI overlay, (**d, h, l, p, t**) lumogallion, DAPI & light overlay. Inserts (marked by an asterisk) show close-ups of individual cells. Magnification X 1000, all scale bars: 20 μm.

Supplementary Tables

Supplementary Table 1 The zeta potential values of native aluminium salts upon dilution into physiological saline (0.9% NaCl) to 0.25mg/mL Al. All measurement were conducted at 25°C and errors are expressed as ± SD of 5 individual replicates.

**Aluminium adjuvant formulation**	**Zeta potential in 0.9% NaCl / mV ± SD**
Alhydrogel®	27.50 ± 2.53
Adju-Phos®	-24.40 ± 3.02

Supplementary Table 2 Intracellular particle sizes (outer diameter, OD) in THP-1 cells co-cultured with 2.5, 25.0, 50.0 or 100μg/mL of either Alhydrogel®, Adju-Phos® or Imject™ Alum for 24 h. Cells were sectioned via agar-paraffin or Spurr resin sectioning. Visualisation of intracellular adjuvant particles for agar-paraffin sectioned THP-1 cells was achieved by fluorescent staining with lumogallion and for TEM by staining with 2% uranyl acetate. Particle sizes were determined by use of the Cell^{D} software suite (Olympus) and mean particle size ± SD are shown (n = 17). Averaging of intracellular particulates across all [ABA]s was performed with n = 340[(1)], 137[(2)] & 93[(3)] for Alhydrogel®, Adju-Phos® and Imject™ Alum, respectively.

**[ABA] / μg/mL**	**Lumogallion-reactive intracellular particle size (OD) / μm (*n* = 17)**		
	Alhydrogel®	Adju-Phos®	Imject™ Alum
2.5	1.01 ± 0.31	1.34 ± 0.31	2.11 ± 0.78
25.0	0.97 ± 0.21	1.31 ± 0.19	1.93 ± 0.59
50.0	0.98 ± 0.21	1.34 ± 0.15	1.86 ± 0.74
100.0	1.10 ± 0.22	1.26 ± 0.23	1.54 ± 0.38
All [ABA]s	0.96 ± 0.19 [(1)]	1.31 ± 0.22 [(2)]	1.90 ± 0.81 [(3)]
**[ABA] / μg/mL**	**Intracellular particulate size as determined by TEM (OD) / μm (*n* = 17)**		
	Alhydrogel®	Adju-Phos®	Imject™ Alum
50.0	0.91 ± 0.14	1.09 ± 0.25	1.21 ± 0.27

Supplementary Table 3 Specifications of the filter membranes used in this study

Filter	Max pore size (µm)	Membrane material	Max. pressure (bar)	Filtration area (cm^2)
Millex – SV (Millipore, UK)	5.60	PVDF	5.2	3.9
Chromacol ECONOFIL (Thermo Scientific, UK)	2.70	PTFE	Not specified	Not specified
Millex-GP express (Millipore, UK)	0.25	PES	10.0	4.5

Supplementary Methods

Graphite furnace atomic absorption spectroscopy of aluminium adjuvant filtrates

Experimental values were derived from the means of three machine replicates per sample provided that the relative standard deviation (RSD) did not exceed 10%. In the event that RSD values exceeded acceptable limits a fresh experimental sample was re-analysed using this method. All absorbance peaks underwent Zeeman background correction through the application of a longitudinal Zeeman-effect background corrector supplied as part of the equipment. Data is expressed as the percentage recovery of aluminium within each filtrate relative to the total Al content within the R10 medium post vaccine administration, where n = 5.

T helper 1 (THP-1) cell culture

All chemicals were from Sigma Aldrich, UK, unless otherwise stated. THP-1 cells (ATCC TIB-202, LGC Standards, UK) were cultured in complete R10 medium which consisted of GlutaMAX™ supplemented 25mM HEPES buffered RPMI 1640 medium, containing 10% *v/v* heat-inactivated foetal bovine serum (certified US origin) (both from Fisher Scientific, Invitrogen). 100μg/mL gentamicin was added from a cell culture certified 10mg/mL stock solution in ultrapure water, to prevent microbial growth. Cell suspensions were passaged to a maximum of 1 x 10^6 cells / mL in TC-treated canted and vented T25 flasks, prior to sub-culturing into T75 flasks (both from VWR, Corning®). All cells were cultured at 37°C in a humidified atmosphere, containing 5% CO_2. Cells were counted using a haemocytometer and their viability confirmed (prior to the addition of ABA), using the Trypan blue exclusion test.

Cytotoxicity assay (Live/dead staining)

Cells designated as dead cell controls were terminated using 70% MeOH for 10 minutes before washing, re-suspension and application to the plate. Prior to plating, estimates of cell viability in both the positive and negative cellular controls were obtained using the trypan blue exclusion method. In all cases positive (live) and negative (dead) cells controls had an estimated viability of >95% & <5% respectively via this method. A solution containing 1µM calcein acetoxymethyl (AM) ester and 5µM ethidium homodimer-1 (EthD-1) in a total volume of 10mL PBS (100µL per well) was administered to all aluminium treatment wells plus control wells containing no cells.

Triplicates of positive live and negative dead controls (+ cells) were exposed to either 1µM calcein AM or 5µM EthD-1 to represent the maximal and minimal fluorescence at 530 and 645nm respectively so that the percentage of live/dead cells in treated samples could be calculated. Plates were subsequently incubated *sine lumine* at ambient temperature (25ºC) for 45 min to allow development. The fluorescence intensity of each well was measured at 530 and 645nm using a GloMax®-Multi+ microplate multimode reader (Promega, UK). Readings were corrected for background fluorescence (PBS blank) and any contribution from the adjuvant and culture medium via subtraction of controls values (- cells) from respective treatments prior to analysis. Results represent the average % mortality of three individual replicates.

Agar-paraffin embedding of THP-1 cells

The preparation and staining of isolated THP-1 cell sections was performed using established methods described elsewhere[16]. Briefly, PFA fixed THP-1 cells co-cultured in the absence or presence of an ABA were pre-embedded into 5% *w/v* molten agar in BEEM® capsules (Elektron, Technology, Agar Scientific) and the blocks dehydrated fully through a graded ethanol series from 30 – 100% *v/v*. For paraffin embedding, the resultant dehydrated agar-cell blocks were cleared by immersion into Histo-Clear (National Diagnostics, USA) for 20 min, with one change of fresh Histo-Clear half way through. Blocks were subsequently infiltrated with paraffin wax maintained at 60°C, for 35 min. 2µm cell sections were prepared by use of a Leica RM2025 rotary microtome and the agar-cell sections were rehydrated back into ultrapure water via reverse infiltration, prior to staining.

Modified agar-paraffin protocol for the preparation of THP-1 cells co-cultured with Imject™ Alum.

The agar-paraffin embedding protocol employed for the sectioning of THP-1 cells necessitated modification for their successful sectioning in the presence of Imject™ Alum. Upon the addition of molten agar to fixed cells, the centrifugation phase used to pellet the block was found to produce dense deposits of the ABA upon final sectioning at 2 μm. As a result, staining of the rehydrated sections with lumogallion for 24 h produced excessive extracellular fluorescence of the ABA. This was particularly problematic at the highest concentrations of 50.0 and 100.0 μg/mL of Imject™ Alum. Therefore, following the addition of molten agar to cells and prior to setting, cell treatments were vortexed at high speed to suspend the cells throughout the block. This was found to considerably reduce extracellular ABA material deposited, allowing for individual cells to be distinguished via DAPI-nucleic staining.

Spurr-resin embedding of THP-1 cells

THP-1 cells for resin embedding were dehydrated as above and then gradually infiltrated with Spurr resin. Fresh Spurr resin was prepared by sequentially weighing 13g NSA (nonenyl succinic anhydride, R1054), 5g ERL 4221 (3,4-epoxycyclohexylmethyl 3,4-epoxycyclohexyl carboxylate), 3g DER (diglycidyl ether of polypropylene glycol, R1074) and 0.2g S-1 (DMAE, dimethylaminoethanol, R1067) (Agar Scientific, UK) into a disposable polyethylene cup. Once all the components were added they were subsequently mixed slowly by use of a

variable speed electric stirrer for a maximum of 15 min. Infiltration of agar-cell blocks was achieved gradually by first transferring the blocks into 3:1 dry ethanol : Spurr resin for 2 h, 1:1 dry 100% ethanol : Spurr resin for 2 h and finally 1:3 dry 100% ethanol overnight, all at ambient temperature. Full infiltration of agar-cell blocks was achieved by performing three changes of fresh Spurr resin over 8 h after which blocks were arranged into moulds and polymerised for a minimum of 16 h in a resin oven at 60°C. Polymerised resin blocks were sectioned at 100nm by use of an automated Leica ultracut UCT ultramicrotome, equipped with a 45° Diatome diamond knife (30-US, Electron Microscopy Sciences).

Electron micrograph acquisition

Samples for TEM were viewed on a JEOL 1230 transmission electron microscope operated at 100.0 kV (spot size 1), equipped with a Megaview III digital camera from Soft Imaging Systems (SIS). An activated field emission of 10µA was used which increased the standing current to 67 – 68µA during operation. Images were obtained on the iTEM universal TEM imaging platform software. Measurements of intracellular ABA particles were made using the Cell^{D} software package and the final editing of electron micrographs for publication was achieved using Photoshop (Adobe systems Inc. USA).

Statistical analyses

Data were analysed for statistical significance using Graph Pad Prism software. ANOVA with repeated measures followed by Tukey post hoc tests were used where multiple

comparisons were required. Where comparisons between two related samples were required a paired t-test was performed. Comparisons between two independent samples were performed using an unpaired t-test. An alpha level of $P \leq 0.05$ (*) was considered statistically significant.
